# A comparison of Knowledge, attitude and practice (KAP) of nurses on nursing Post-stroke dysphagia patients between iii-A and ii-A hospitals in China: a propensity score-matched analysis

**DOI:** 10.1186/s12912-022-00950-x

**Published:** 2022-06-29

**Authors:** Shumin Deng, Xiaolan Mao, Xianmei Meng, Liping Yu, Fei Xie, Guiling Huang, Zhizhou Duan

**Affiliations:** 1 Department of Clinical Data Center, The Third Affiliated Hospital of Sun Yat-Sen University, Guangzhou, People’s Republic of China; 2 Neurology department, Zhongnan Hospital of Wuhan University, Wuhan, China; 3 Wuhan University School of Nursing, Wuhan University, Wuhan, China; 4 Preventive Health Service, Jiangxi Provincial People’s Hospital, The First Affiliated Hospital of Nanchang Medical College, Nanchang, Jiangxi China

**Keywords:** KAP, Post-stroke dysphagia, Hospital ranking, Propensity score matched

## Abstract

**Background:**

Whether knowledge, attitude and practice of nurses on nursing post-stroke dysphagia patients varies between different ranking hospitals is still unknown. This study aimed to compare the knowledge, attitude and practice level of nurses on nursing post-stroke dysphagia patients between iii-A and ii-A hospitals in China.

**Design:**

A cross-sectional study design was used.

**Methods:**

Data were collected from eighteen hospitals in Wuhan, Hubei in May–July 2020, and a total of 824 nurses were recruited by convenient sampling. After propensity score matching, 205 participants in iii-A hospitals were matched with 205 participants in ii-A hospitals.

**Results:**

There were no statistically differences in the socio-demographic characteristics between two groups after propensity score matching. Before matching, the regression coefficients between hospital ranking and knowledge, attitude, practice were -0.415, -0.718 and -1.855, respectively. After matching, the coefficients changed to -0.394, -0.824 and -1.278. Nurses from iii-A hospitals had higher knowledge and attitude scores than nurses from ii-A hospitals, but no significant practice scores difference was observed between various rankings of hospitals.

**Conclusions:**

The KAP of nurses on nursing post-stroke dysphagia patients were different in iii-A and ii-A hospitals. Administrators should strengthen management, provide more learning resources and trainings to meet nurses’ needs about methods to deal with and recognize dysphagia, so as to further improve the quality of post-stroke dysphagia management.

## Background

Stroke is a leading cause of disability and death in adults globally. There were 6.55 million deaths and 143 million disability-adjusted life-years (DALYs) due to stroke in 2019 worldwide, and nearly one-third of that occurred in China [1, 2]. Difficulty in swallowing is a common condition after stroke, which called post-stroke dysphagia (PSD). Previous studies suggested that up to 80% had swallowing problems of whom half would be symptomatic after ictus, and the prevalence of dysphagia in acute stroke has been reported ranging between 28 and 65% around the world [3, 4]. PSD is associated with multiple unfavorable outcomes including aspiration pneumonia, modified diet or reliance on tube feeding, dehydration and malnutrition. Furthermore, compares to non-cases, dysphagia increased the mean length of hospital stay by 3.8 days, total inpatient cost by 48%, and 1.7 times mortality [5, 6], hence early recognition of dysphagia and management in the stroke patients should be prioritized.

PSD management primarily aims to reduce aspiration and to ease swallowing difficulties, and measures such as altering posture, modifying food and changing feeding strategies are often used independently or together [5]. In current clinical guidelines of the UK, Europe, Canada, the USA, and Australia, the recommendation to perform a dysphagia screen after stroke was classed as consensus-based [7, 8]. Besides, “Expert Consensus on the Evaluation and Treatment of dysphagia in China (2017 edition)” summarized several tools for dysphagia screen, such as eating assessment tool (EAT-10), Toronto bedside swallowing screening test (TOR-BSST), etc., and suggested that a collaborative team approach was ideal for the PSD confirmation [9, 10]. Strong evidences proved that nurses played a key role in the early detection and management of patient with PSD, and if nurses are trained to master PSD screening or management skills, not only the number of accurate screen performed will increase, but inpatient death and chest infections will decrease [11–13]. Previous studies have revealed that knowledge, attitude and practice (KAP) play a significant role in personal experience and effect behaviors [14, 15]. Consequently, the level of knowledge, attitude and practice of nursing PSD patients among nurse staff will determine the quality of dysphagia care and might be of great significant to deliver better patient outcomes.

In general, past PSD relevant researches have mostly focused on screening tools available to nurses and dysphagia treatments. There are few studies on the nurses’ knowledge, attitude and practice of PSD no matter in China or abroad [16–18]. Nurses from diverse cultures are certain to face different clinical problems and medical environment, thus conducting researches on nurses’ KAP of PSD nursing based on different cultural backgrounds is needed. Within the health care infrastructure in China, hospitals were divided into three levels (i, ii, iii) based on the scale, medical safety, service quality and other capacities, with three ranks (A, B, C) for each level [19, 20]. The primary care facilities (level i) are mostly township hospitals with several attending physicians who had general medical training and the secondary care facilities (level ii) are mostly county hospitals with 3–4 attending physicians who had some specialized training per specialty. Tertiary care hospitals (level iii) are equipped with experienced specialist team in each specialty, and considered to be top-ranked with best health care capacity and quality [21].

Although referral system has been implemented for many years in China, it is very common for patients to bypass primary hospitals and seek care at higher level hospitals because of lack of confidence in lower level hospitals [22]. Some researches argued that the inefficient referral processes in China resulted in overcrowded tertiary hospitals, and the care quality provided by those institutions might be questionable and not definitely better than that provided by lower level hospitals [23]. There is litter empirical evidence to determine whether nurses engaged in stroke related nursing work in level iii hospitals have higher level of KAP than those in level ii hospitals. To fill the gap in the literature, this study aims to compare the KAP level of nurses on nursing PSD patients between iii-A and ii-A hospitals in China using the propensity score matching (PSM) method, which is often used in clinical researches rather than epidemiology, and provide basis for carrying out adaptively targeted nursing training, so as to improve nursing quality and reduce adverse outcomes in stroke patients.

## Methods and material

### Study design and setting

A cross-sectional survey was conducted in eighteen hospitals in Wuhan, Hubei from May 2020 to July 2020. Wuhan is the capital of Hubei province, the only subprovincial city and megacity in central China, with 9.16 million people at the end of 2020. According to the data issued by Hubei Provincial Statistics Bureau, there were more than 300 hospitals with nearly 45 thousand enrolling nurses, providing more than 40 million person-times services in 2020.

### Inclusion criteria

Nurses were included if they meet all of the following criteria at the same time: 1) nurses with qualified certification; 2) work in Neurology, Neurosurgery, Rehabilitation, or Geriatrics department; 3) willing to sign informed consent. Nurses would be excluded if they meet as least one of the following criteria: 1) intern nurses; 2) nurses undertaking a ward rotation.

### Sample size and sampling technique

The sample size was calculated using cross-sectional survey formula, $\mathrm{n}={(\frac{{Z}_{1-\alpha /2}\times \sigma }{\delta })}^{2}$, where ${Z}_{1-\alpha /2}=1.96$ at 95%confidence level. Since there are few published data that show the knowledge, attitude and practice of PSD nursing among nurses in China, standard deviation $\sigma$ was set to 8.59 in reference to Sun et al. [18], and tolerant error $\delta$ was set to 10% $\sigma$. There of 10% of the sample size would add for the non-response rate so that it would be a 427 study subjects at least.

Convenient sampling methods was applied and Wenjuanxing platform, a professional software for data collection questionnaire, was used to distribute questionnaires.

### Data collection instruments and operational definitions

Participants were required to complete a self-administered questionnaire including socio-demographic characteristics, PSD patient nursing knowledge, attitude and practice. Socio-demographic characteristics included age, sex, educational level, work experience (years), hospital ranking, and clinical department. Knowledge, attitude and practice of nurses on post-stroke dysphagia were measured by a scale constructed by Mao et.al, which was proved to be suitable for Chinese nurses [24].

Nursing knowledge consists of 14 questions in yes/no/don’t know format, such as “cough was a symptom of dysphagia when stroke patients were drinking or eating”, “prolonged eating time was a symptom of dysphagia”, etc., and 1 point for correct answer, 0 for others. The total knowledge score (0–14 points) was categorized as good if the score was between 80 and 100% (11.2–14 points), moderate if the score was between 60 and 79% (8.4–11.1 points), and poor if the score was less than 60% (< 8.4 points).

Attitude was measured by 8 items of questions, e.g. “it’s doctors’ duty to screen dysphagia”, and a 5-point Likert Scale was applied, with questions scaled form “strongly disagree” to “strongly agree”. The total attitude score (8–40 points) was categorized as good if the score was between 80 and 100% (32–40 points), moderate if the score was between 60 and 79% (24–31.9 points), and poor if the score was less than 60% (< 24 points).

Practice was measured 10 items of questions, e.g. “do you present the right eating way of stroke patients towards their families”, and the answers were designed to utilize 5-point Likert Scale from “never” to “always”. The total practice score (10–50 points) was categorized as good if the score was between 80 and 100% (40–50 points), moderate if the score was between 60 and 79% (30–39.9 points), and poor if the score was less than 60% (< 30 points).

Higher scores for each section would indicate higher levels of nursing knowledge, attitude and practice. The Cronbach’s alpha values of each of the 3 sections are 0.75, 0.73, and 0.89, respectively. Wenjuanxing platform, a professional software for data collection questionnaire, was used to distribute questionnaires. Finally, 867 questionnaires were distributed, and 824 were deemed valid, indicating an effect rate of 95.0%.

### Data quality assurance

Data collection was undertaken by three nursing masters from Wuhan University. Data collectors received a half day training on concerning the questionnaire, including the objective of this research, the ways of questionnaire distribution, data check and confirmation, etc. Besides, Wenjuanxing platform was well used to set maximum and minimum values during data entry, and correct some logic errors.

### Data processing and statistical analysis

In this study, the scores of nursing knowledge, attitude and practice were considered as the primary outcomes. By using propensity score-matched analysis, the basal characteristics between nurses in different ranking hospitals could be balanced. Thus, propensity score-matched analysis was applied for the primary analysis in the study. Matching was carried our using the 1:1 nearest neighbor method without replacement under a logit model, which yielded 205 participants in iii-A hospital group matched with 205 participants in ii-A hospital group.

Descriptive data were presented as n(%) and mean ± standard deviation. Chi-square tests were executed for comparison of categorical variables. Multiple linear regression analysis was performed in the matched sample. All data were analyzed using R version 4.0.1. A two-side P-value < 0.05 was considered statistically significant.

### Ethics statement

The study was approved by the Ethnic of “Wuhan university”, and the grant number was “2020YF0037”.

## Results

### Univariate analysis

The socio-demographic characteristics and KAP of participants were presented in Table 1. The overwhelming majority of nurses (97.2%) were women and the mean age of them was 30.56 years, ranging from 19 to 55. Most of the nurses (73.7%) had a bachelor’s degree and around 27.2% of them had been working for more than one decade. Nearly three quarters of the participants come from iii-A hospitals, besides, most of them engaged in neurology and neurosurgery. The scores of knowledge, attitude, and practice varied from 0–14, 8–40, 10–50, with an average of 12.32, 30.27, and 36.27, respectively, indicating good knowledge, moderate attitude and practice.

**Table 1:** Socio-demographic characteristics and KAP of participants (*N* = 824)

Characteristic	Number (N/%)	Knowledge	Attitude	Practice
Total	824	12.32 ± 1.99	30.27 ± 4.20	36.27 ± 7.73
Age (years)	30.56 ± 6.39			
19–30	491 (59.6)	12.23 ± 2.09	30.05 ± 4.29	35.75 ± 7.90
31–40	269 (32.6)	12.43 ± 1.86	30.63 ± 4.20	36.75 ± 7.51
41–55	64 (7.7)	12.47 ± 1.70	30.47 ± 3.42	38.30 ± 6.84
Sex				
Women	801 (97.2)	12.32 ± 1.99	30.27 ± 4.16	36.27 ± 7.74
Men	23 (2.8)	12.26 ± 2.18	30.39 ± 5.57	36.35 ± 7.24
Education level				
High school or lower	200 (24.3)	11.90 ± 2.17	29.33 ± 4.17	34.67 ± 7.86
Bachelor degree	607 (73.7)	12.45 ± 1.91	30.56 ± 4.12	36.75 ± 7.66
Master degree or higher	17 (2.1)	12.47 ± 1.81	31.24 ± 5.94	38.18 ± 5.71
Experience (years)				
1–5	332 (40.3)	12.18 ± 2.17	29.78 ± 4.29	36.13 ± 7.92
6–10	268 (32.5)	12.34 ± 1.96	30.56 ± 4.25	35.87 ± 7.80
11–15	113 (13.7)	12.30 ± 1.81	30.58 ± 4.18	36.12 ± 7.76
16–35	111 (13.5)	12.68 ± 1.60	30.74 ± 3.73	37.83 ± 6.79
Hospital ranking				
iii-A	613 (74.4)	12.39 ± 1.94	30.48 ± 4.18	36.87 ± 7.63
ii-A	211 (25.6)	12.09 ± 2.10	29.67 ± 4.23	34.55 ± 7.76
Clinical department				
Neurology	446 (54.1)	12.50 ± 1.74	30.54 ± 4.13	36.72 ± 7.64
Neurosurgery	145 (17.6)	11.95 ± 2.33	29.49 ± 4.55	34.22 ± 7.00
Rehabilitation	54 (6.6)	12.94 ± 1.75	30.83 ± 3.28	40.81 ± 7.08
Geriatrics	179 (21.7)	11.96 ± 2.24	30.08 ± 4.27	35.45 ± 8.05

**Fig. 1:**
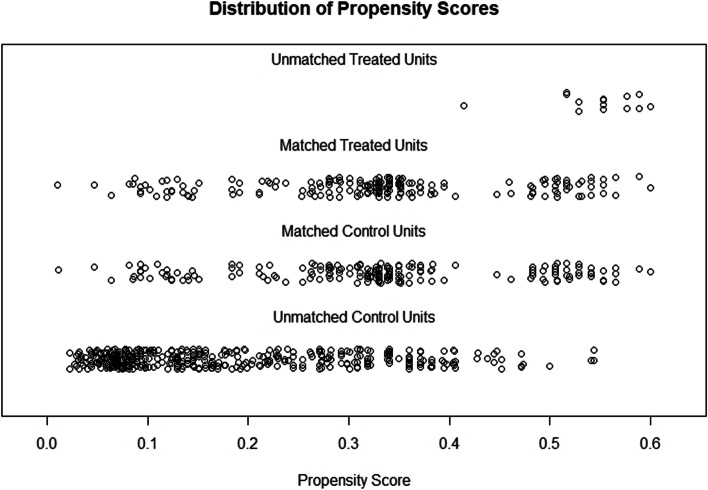
The distribution of propensity score before and after PSM analysis

**Fig. 2:**
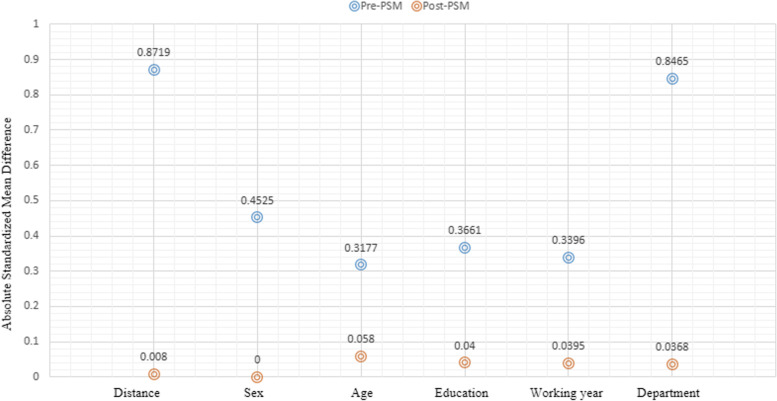
The absolute standardized mean difference before and after PSM analysis

Prior to propensity score matching, 613 nurses in iii-A hospitals and 211 in ii-A hospitals were identified. Socio-demographic characteristics were all significantly different between two groups with regard to age, sex, educational level, work experience and clinical department (Table 2). After propensity score matching, 205 nurses in iii-A hospitals were matched to 205 nurses in ii-A hospitals with statistically identical socio-demographic characteristics (*p* > 0.5, Table 2). Approximately two-thirds of the discharges in each matched group were aged between 19–30 years and had a bachelor’s degree. Meanwhile, the sum distance of the absolute standardized mean difference had decreased from 0.87 to 0.008, indicating that the “nearest” method of PSM analysis was appropriate (Figs. 1 and 2).

**Table 2:** The socio-demographic characteristics of participants before and after PSM between iii-A and ii-A hospitals

Variables	Before PSM analysis (*N* = 824)		χ^2^	*P*	After PSM analysis (*N* = 410)		χ^2^	*P*
	iii-A (*N* = 613)	ii-A (*N* = 211)			iii-A (*N* = 205)	ii-A (*N* = 205)		
Age (years)			12.51	0.002			0.74	0.691
19–30	344(70.1)	147(29.9)			136(49.1)	141(50.9)		
31–40	215(79.9)	54(20.1)			61(53.0)	54(47.0)		
41–55	54(84.4)	10(15.6)			8(44.4)	10(55.6)		
Sex			5.61	0.018			0	1.000
Women	591(73.8)	210(26.2)			1(50.0)	1(50.0)		
Men	22(95.7)	1(4.3)			204(50.0)	204(50.0)		
Education level			23.57	< 0.001			0.55	0.761
High school or lower	124(62.0)	76(38.0)			63(47.4)	70(52.6)		
Bachelor degree	473(77.9)	134(22.1)			141(51.3)	134(48.7)		
Master degree or higher	16(94.1)	1(5.9)			1(50.0)	1(50.0)		
Experience (years)			13.14	0.004			0.12	0.989
1–5	240(72.3)	92(27.7)			83(49.1)	86(50.9)		
6–10	192(71.6)	76(28.4)			77(50.3)	76(49.7)		
11–15	83(73.5)	30(26.5)			32(51.6)	30(48.4)		
16–35	98(88.3)	13(11.7)			13(50.0)	13(50.0)		
Clinical department			72.17	< 0.001			1.78	0.619
Neurology	282(63.2)	164(36.8)			160(50.3)	158(49.7)		
Neurosurgery	115(79.3)	30(20.7)			23(43.4)	30(56.6)		
Rehabilitation	49(90.7)	5(9.3)			8(61.5)	5(38.5)		
Geriatrics	167(93.3)	12(6.7)			14(53.8)	12(46.2)

### Linear regression analysis

**Fig. 3:**
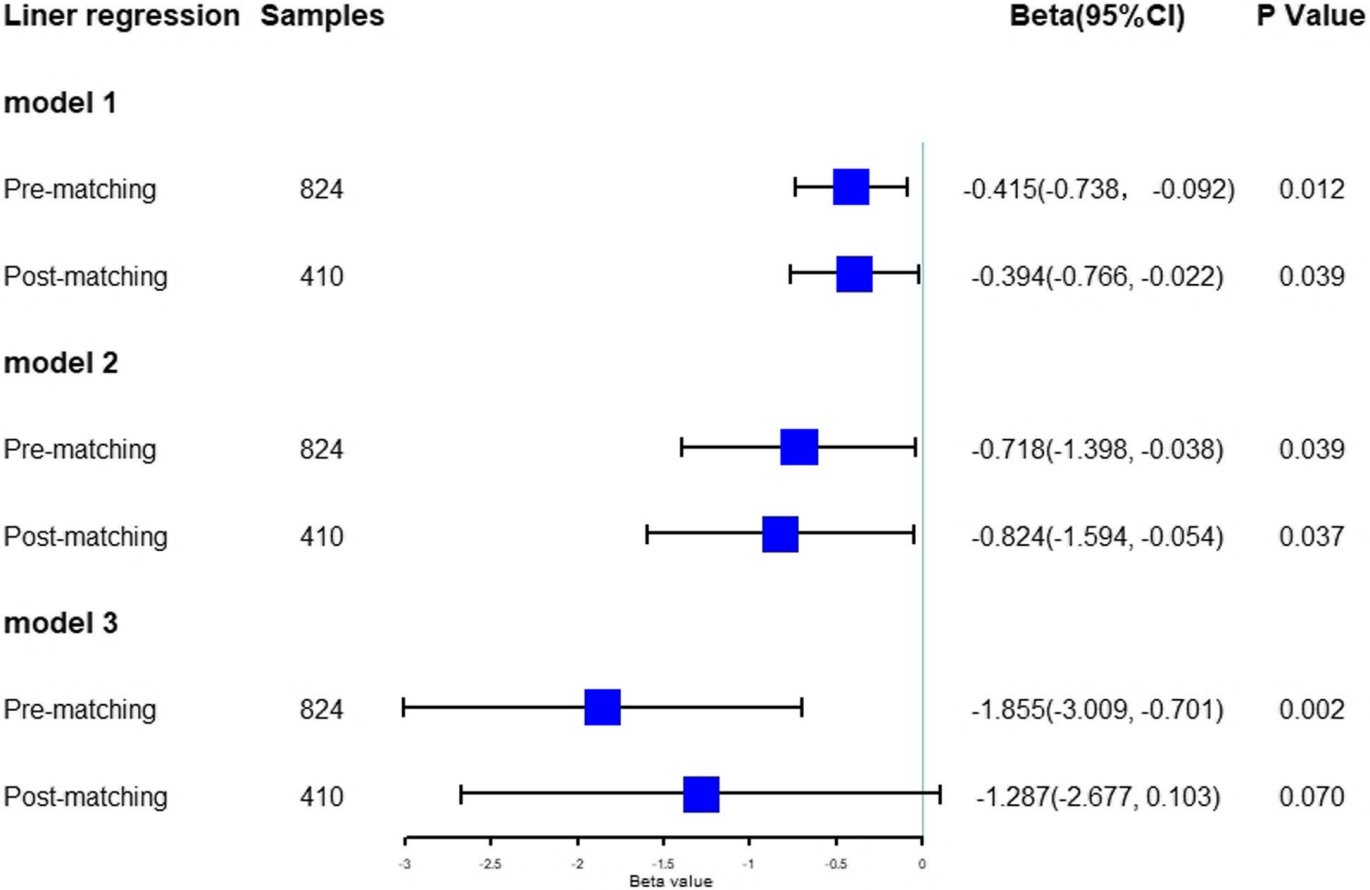
The effect of hospital ranking on knowledge, attitude, and behavior before and after PSM analysis. Note: Model 1: outcomes-knowledge, factors-basic characteristics + hospital ranking. Model 2: outcomes-attitude, factors-basic characteristics + hospital ranking + knowledge. Model 3: outcomes-behavior, factors-basic characteristics + hospital ranking + knowledge + attitude

Figure 3 showed the results of linear regression analysis adjusting for nurse socio-demographic characteristics before and after propensity score matching. Before PSM, the regression coefficients between hospital ranking and knowledge, attitude, practice were -0.415, -0.718 and -1.855, respectively. After matching, the coefficients between hospital ranking and knowledge, attitude changed to -0.394 and -0.824 with statistic significantly, indicating both the scores of knowledge, attitude in iii-A hospital group were higher than those in ii-A hospital group. While the effect of hospital ranking on practice scores changed to be no significance (β = -1.287, *p* = 0.070), indicating that there were no statistically significant practice scores difference observed between iii-A hospitals and ii-A hospitals.

## Discussion

To our knowledge, this is the first propensity score-matched analysis examining the effects of hospital ranking on nurses’ knowledge, attitude and practice levels about nursing PSD patients. Propensity score matching combined with regression analysis was widely used in clinical researches for effectiveness or costs comparison [25, 26], which was of significant importance to minimize the effect of selection bias on the study hypothesis. This study demonstrated the benefits of PSM approach, for avoiding overestimating or underestimating the effects of hospital rankings on knowledge, attitude and practice scores compared with traditional linear regression analysis.

Generally, dysphagia occurs insidiously and nurses are the first professionals to recognize the signs and symptoms of PSD, so it is critical for nurses being fully aware of PSD [27]. The average scores of knowledge, attitude, and practice in this study were 12.32, 30.27, and 36.27, respectively. Compared with the survey conducted in Beijing (2020) [18], nurses’ KAP on PSD nursing in this study were promising but nurses’ knowledge, attitude, practice still urgently needed to be strengthened for better care quality. A multicenter survey conducted in Sichuan province [28] revealed that hospital level was one of the main factors influencing geriatric dysphagia-related knowledge scores. Similar to that, our study found that nurses from iii-A hospitals had higher level of knowledge and attitude than nurses from ii-A hospitals. Nurses in higher ranking hospitals have more opportunities to access richer learning resources and have a wider range of learning pathways, such as receive training courses, continuing education and participating in academic forums [28]. Besides, learning and training in the care of PSD are effective ways of acquiring knowledge and improving attitude of dysphagia management [18]. From this sense, providing more learning resources and more dysphagia-specific trainings for nurses in ii-A hospitals may help to narrow the gap between them and nurses in higher ranking hospitals.

It’s noted that no statistically significant practice scores difference was observed between various rankings of hospitals. Nurses from iii-A hospitals did not score higher on PSD nursing practice, although they had significantly greater PSD nursing knowledge and more positive attitude. There is some evidence to support this as practice was not found to have significant correlation with attitude or knowledge [29, 30]. According to the KAP theory, the changes of human behavior were divided into three successive processes: the acquisition of knowledge, the generation of attitudes and the formation of behavior. PSD nursing knowledge was an intellectual prerequisite for performing correct nursing behavior, and knowledge is expected to have a positive and indirect effect on practice (behavior) by changing attitude [31–33]. No practice score difference was found between two groups in this study might be due to not enough knowledge and attitude scores to trigger behavior changes. Another more compelling reason might lie in work environment. Given patients’ demand for better care quality and the nonfunctional referral system, more and more patients seek care from iii-A hospitals, even for minor conditions. As nurse-patient ratio increases, nurses’ workload become heavier and care quality deteriorated [34]. Although nurses in iii-A hospitals have higher level of knowledge and attitude, it is hard to make behavioral change.

The results demonstrated substantial scope for improving nurses’ knowledge, attitude and practice on PSD nursing. Governments of all levels in China need to continuously make effort to effectively implement referral system and differentiate services provide by hospitals so that nurses’ workload in iii-A hospitals can be released from most primary care services, then nurses can improve their care quality. Meanwhile, administrators in ii-A hospitals should strengthen management, provide more training and learning opportunities for nurses working with stroke patients on methods to deal with and recognize dysphagia, such as appropriate swallowing posture and maneuvers, and diet modifications [35, 36]. In clinical practice, experienced nurses can be paired with new nurses to decrease knowledge and competency gaps between them, and enhance their skills appropriate to their role in the pathway, so as to achieve overall improvement in the quality of the management of PSD patients [28, 37].

## Limitations

This study has some limitations. First, the study was limited by cross-sectional design that could not infer causality among the variables or account for unknown confounders. Second, the data were collected by self-reporting, which might result in information bias owing to concealment. Third, participants were sampled from Wuhan by using convenient sampling, the results might not represent the whole situation of hospitals in China.

## Conclusion

This is the first study to compare the KAP of nurses on PSD between iii-A and ii-A hospitals. The results showed that participants had a promising KAP regarding dysphagia. Although nurses from iii-A hospitals were found to have better knowledge and attitude than nurses from ii-A hospitals, more efforts were still needed to increase the KAP of nurses about PSD nursing. Administrators should improve referral system and strengthen management, provide more learning resources and trainings to meet nurses’ needs about methods to deal with and recognize dysphagia, so as to further improve the quality of PSD management. Further studies on PSD nursing are warranted to conduct in more areas with large samples to increase our understandings of this important filed.

## Data Availability

The datasets generated during and analyzed during the current study are not publicly available due to privacy but are available from the corresponding author on reasonable request.

## Abbreviations

DALYs: Disability-adjusted life-years
KAP: Knowledge, attitude and practice
PSD: Post-stroke dysphagia
PSM: Propensity score matching
